# Diagnostic accuracy of whole-body MRI versus standard imaging pathways for metastatic disease in newly diagnosed non-small-cell lung cancer: the prospective Streamline L trial

**DOI:** 10.1016/S2213-2600(19)30090-6

**Published:** 2019-06

**Authors:** Stuart A Taylor, Sue Mallett, Simon Ball, Sandy Beare, Gauraang Bhatnagar, Angshu Bhowmik, Peter Boavida, John Bridgewater, Caroline S Clarke, Marian Duggan, Steve Ellis, Robert Glynne-Jones, Vicky Goh, Ashley M Groves, Ayshea Hameeduddin, Sam M Janes, Edward W Johnston, Dow-Mu Koh, Sara Lock, Anne Miles, Stephen Morris, Alison Morton, Neal Navani, Alfred Oliver, Terry O'Shaughnessy, Anwar R Padhani, David Prezzi, Shonit Punwani, Laura Quinn, Hameed Rafiee, Krystyna Reczko, Andrea G Rockall, Peter Russell, Harbir S Sidhu, Nicola Strickland, Kathryn Tarver, Jonathan Teague, Steve Halligan, Streamline investigators, Ruth Evans, Khawaja Shahabuddin, Revanth Jannapureddy, Tina Mills-Baldock, Kishor Barhate, Zoltan Nagy, Sherif Raouf, Akosa Aboagye, Girija Anand, Rommel Butawan, Elizabeth Hadley, Adesewa Onajobi, Mohamed A Thaha, Tanjil Nawaz, Catherine Norman, Nathalie Rich, Sidra Tulmuntaha, Shafi Ahmed, Louise Lim, Fiona McKirdy, Jenna Couture, Shahanara Ferdous, Payal Julka, Ali Mohammed, Helen Pardoe, Sanjaya Wijeyekoon, Katherine Van Ree, Dominic Blunt, William Ricketts, Marie Jackson, Clive Kay, Andy Lowe, Janet McGowan, Amjad Mohammed, Jon Robinson, Lara Curry, Sasithar Maheswaran, Subramanian Ramesh, Pippa Riddle, Shaki Balogun, Yvonne Campbell, Nelesh Jeyadevan, Aji Kavidasan, Imogen Locke, Tuck-Kay Loke, Ibiyemi Olaleye, Clare Collins, Elizabeth Green, Colm Prendergast, Thida Win, Amy Davis, Lyn Blakeway, Sofia Gourtsoyianni, Adrian Green, Christian Kelly-Morland, Sahar Naaseri, John O'Donohue, David Snell, Dorothee Boisfer, Keyury Desai, Balinder Hans, Sophia Hans, Eleni Ntala, Adnam Alam, Stephen Burke, Matthew Train, Nishat Bharwani, Gule Hanid, Lesley Honeyfield, Tina Stoycheva, Uday Patel, Farid Bazari, Helen Beedham, Jane De Los, Reyes Lauigan, Priya Limbu, Nicola Lucas, Sally O'Connor, Anita Rhodes, Laletha Agoramoorthy, Martha Handousa, Abel Jalloh, Stefania Stegner, Shanna Wilson, David Birch, Suzanne Chukundah, Priscilla Phiri, Raj Srirajaskanthan, Eleni Karapanagiotou, Daniel Smith, Ferrial Syeed, Chloe van Someren, Rudi Borgstein, Jamila Roehrig, David Chao, Lorraine Hurl, Andrew Gogbashian, Andre Nunes, Ian Simcock, James Stirling, Richard Beable, Maureen Furneaux, Nicola Gibbons, Antony Higginson, Howard Curtis, Kitrick Perry, Anita Amadi, Heather Hughes, Prital Patel, Gary Atkin, Colin Elton, Stephen Karp, Lisa Woodrow, Dominic Yu, Sajid Khan, Alistair Rienhardt, Pooja Datt, Rajapandian Ilangovan, Ian Jenkins, Saba Mahmud, Teresa Light, Joanne Kellaway, Ann O'Callaghan, William Partridge, Amelia Daniel, Ugo Ekeowa, Michael Long, Erica Scurr, Veronica Morgan, Nina Tunariu, Elizabeth Chang, Laura Hughes, Ellice Marwood, Katie Prior, Meena Reddi, Kara Sargus, Abby Sharp, Teresita Beeston, Elizabeth Isaac, Adoracion Jayme, Jagadish Kalasthry, Wivijin Piga, Farzana Rahman, Shraddha Weir, Aileen Austria, James Crosbie, Alec Engledow, Jonathan McCullogh, Austen Obichere, Kai-Keen Shiu, Christopher Wanstall, Celia Simeon, Amy Smith, Andrew Bateman, David Breen, Liane Davis, Chris Everitt, Alice Johnson, Paul Nichols, Beth Shepherd, Kayleigh Gilbert, Azmina Verjee, Michelle Saull, Jonathan Wilson, Rashidat Adeniba, Veronica Conteh, Sarah Howling

**Affiliations:** a Centre for Medical Imaging, University College London, London, UK; b Cancer Research UK & UCL Cancer Trials Centre, University College London, London, UK; c Institute of Nuclear Medicine, University College London, London, UK; d Lungs for Living Research Centre, UCL Respiratory, University College London, London, UK; e Research Department of Primary Care and Population Health, University College London, London, UK; f Department of Applied Health Research, University College London, London, UK; g Institute of Applied Health Research, NIHR Birmingham Biomedical Research Centre, College of Medical and Dental Sciences, University of Birmingham, Edgbaston, Birmingham, UK; h Barking, Havering, and Redbridge NHS Trust, Romford, UK; i Frimley Park Hospital, Frimley, UK; j Department of Respiratory Medicine, Homerton University Hospital, London, UK; k Department of Radiology, Homerton University Hospital, London, UK; l UCL Cancer Institute, London, UK; m Department of Radiology, Barts Health NHS Trust, London, UK; n Mount Vernon Centre for Cancer Treatment, Mount Vernon Hospital, Northwood, UK; o Department of Cancer Imaging, School of Biomedical Engineering and Imaging Sciences, King's College London, King's Health Partners, London, UK; p Department of Thoracic Medicine, University College London Hospitals, UK; q Department of Radiology, Royal Marsden Hospital, Sutton, Surrey, UK; r Department of Respiratory Medicine, Whittington Hospital, London, UK; s Department of Psychological Sciences, Birkbeck University of London, London, UK; t Department of Respiratory Medicine, Barts Health NHS Trust, London, UK; u Paul Strickland Scanner Centre, Mount Vernon Cancer Centre, Northwood, UK; v Department of Radiology, Guy's & St Thomas' NHS Foundation Trust, London, UK; w Norfolk and Norwich University Hospitals NHS Foundation Trust, Norwich, UK; x Department of Imaging, Hammersmith Hospital, Imperial College Healthcare NHS Trust, London, UK; y Department of Cancer and Surgery, Imperial College London, London, UK; z Department of Respiratory Medicine, Princess Alexandra Hospital NHS Trust, Harlow, UK

## Abstract

**Background:**

Whole-body magnetic resonance imaging (WB-MRI) could be an alternative to multi-modality staging of non-small-cell lung cancer (NSCLC), but its diagnostic accuracy, effect on staging times, number of tests needed, cost, and effect on treatment decisions are unknown. We aimed to prospectively compare the diagnostic accuracy and efficiency of WB-MRI-based staging pathways with standard pathways in NSCLC.

**Methods:**

The Streamline L trial was a prospective, multicentre trial done in 16 hospitals in England. Eligible patients were 18 years or older, with newly diagnosed NSCLC that was potentially radically treatable on diagnostic chest CT (defined as stage IIIb or less). Exclusion criteria were severe systemic disease, pregnancy, contraindications to MRI, or histologies other than NSCLC. Patients underwent WB-MRI, the result of which was withheld until standard staging investigations were complete and the first treatment decision made. The multidisciplinary team recorded its treatment decision based on standard investigations, then on the WB-MRI staging pathway (WB-MRI plus additional tests generated), and finally on all tests. The primary outcome was difference in per-patient sensitivity for metastases between standard and WB-MRI staging pathways against a consensus reference standard at 12 months, in the per-protocol population. Secondary outcomes were difference in per-patient specificity for metastatic disease detection between standard and WB-MRI staging pathways, differences in treatment decisions, staging efficiency (time taken, test number, and costs) and per-organ sensitivity and specificity for metastases and per-patient agreement for local T and N stage. This trial is registered with the International Standard Randomised Controlled Trial registry, number ISRCTN50436483, and is complete.

**Findings:**

Between Feb 26, 2013, and Sept 5, 2016, 976 patients were screened for eligibility. 353 patients were recruited, 187 of whom completed the trial; 52 (28%) had metastasis at baseline. Pathway sensitivity was 50% (95% CI 37–63) for WB-MRI and 54% (41–67) for standard pathways, a difference of 4% (−7 to 15, p=0·73). No adverse events related to imaging were reported. Specificity did not differ between WB-MRI (93% [88–96]) and standard pathways (95% [91–98], p=0·45). Agreement with the multidisciplinary team's final treatment decision was 98% for WB-MRI and 99% for the standard pathway. Time to complete staging was shorter for WB-MRI (13 days [12–14]) than for the standard pathway (19 days [17–21]); a 6-day (4–8) difference. The number of tests required was similar WB-MRI (one [1–1]) and standard pathways (one [1–2]). Mean per-patient costs were £317 (273–361) for WBI-MRI and £620 (574–666) for standard pathways.

**Interpretation:**

WB-MRI staging pathways have similar accuracy to standard pathways, and reduce the staging time and costs.

**Funding:**

UK National Institute for Health Research.

## Introduction

Non-small-cell lung cancer (NSCLC) is the leading cause of cancer related death in the UK, with more than 35 000 deaths annually.^1^ Accurate staging is fundamental for optimal patient outcomes, particularly identification of metastatic disease, because this typically dictates therapeutic strategy. At least 20% of patients who undergo curative lung surgery relapse with undiagnosed metastatic disease (so-called futile thoracotomy),^2^ indicating that the current approach to NSCLC staging is suboptimal. Staging pathways are complex, relying on high technology imaging platforms such as CT, PET-CT, and MRI. In England, for example, the National Institute for Health and Care Excellence (NICE) publishes guidelines that require multiple, sequential imaging tests to complete staging and allow the first treatment decisions to be made.^3^, ^4^ The complexity of staging pathways is due to modalities having variable accuracies across organs at risk for harbouring metastases. Standard pathways are, therefore, time and resource intensive, irradiate patients,^5^ and increase anxiety if protracted.^6^

**Research in context**

**Evidence before this study**

The detection of metastatic disease during non-small-cell lung cancer (NSCLC) staging underpins treatment strategy and is fundamental to optimisation of patient outcomes. Staging pathways rely on high technology imaging platforms such as CT, PET-CT, and MRI, which differ in their diagnostic accuracies across individual organs. Such multimodality staging pathways are complex, resource and time intensive, involve irradiation, and increase patient anxiety. Modern MRI platforms can image the whole body within 1 h, and whole-body MRI (WB-MRI) is advocated as a more accurate, efficient, and safer alternative to multimodality staging pathways. We searched PubMed and Embase (without language restriction) for articles published between Jan 1, 1990, and Sept 30, 2018, using MeSH and full-text search-strings for “cancer”, “neoplasm”, “staging”, “diagnostic accuracy”, “magnetic resonance imaging”, “whole body imaging”, “diffusion magnetic resonance imaging”, “metastasis”, and “lung”. We found several meta-analyses reporting WB-MRI accuracy for lung cancer staging, most suggesting accuracy for metastatic disease is equivalent to, or might exceed standard technologies. All such meta-analyses, however, were limited to metastasis detection in specific end organs, notably bone. Various comparators have been selected but the majority compare WB-MRI with PET-CT, and scintigraphy (in the case of bone metastasis). Most primary studies were small, single site, and explanatory, with WB-MRI interpreted by a few specialised radiologists. They focused on single modality comparisons rather than evaluating real-world, multimodality staging pathways. We found no data regarding how WB-MRI influences the first major treatment decision or staging efficiency.

**Added value of this study**

To our knowledge, this is the largest prospective multicentre trial to date comparing the diagnostic accuracy of WB-MRI staging pathways to standard staging in patients newly diagnosed with NSCLC. We used a pragmatic trial design to better test pathway performance in routine clinical practice and investigated staging pathway efficiency in terms of test number, time to completion, and costs. We also contemporaneously tested the effect of alternative staging pathways on the nature and timing of the first major treatment decisions. Patient outcomes were followed-up after 12 months to better evaluate pathway accuracy at the time of initial staging. We found both pathways had similar accuracies for identifying patients with metastatic disease and the nature of the first major treatment decision was similar. WB-MRI was more efficient and reduced the time to staging completion and costs.

**Implications of all the available evidence**

WB-MRI staging pathways have similar accuracy to current standard staging pathways, resulting in the same treatment decisions. However, they are more efficient and reduce time to complete staging and costs. WB-MRI is, therefore, more suitable for staging in routine clinical practice. Future research should investigate the utility of WB-MRI treatment response assessment and cancer surveillance after curative treatments.

Modern MRI scanners can image the entire body within 1 h, and whole-body MRI (WB-MRI)—which typically scans from the head to mid-thigh—is a potentially more accurate and safer alternative to standard multimodality staging pathways. WB-MRI could also accelerate staging, thereby increasing efficiency by reducing additional tests, staging time, and costs. Meta-analyses suggest accuracy of WB-MRI in detecting metastatic disease for metastatic disease is equivalent to, or might exceed, standard technologies,^7^, ^8^, ^9^, ^10^, ^11^, ^12^, ^13^, ^14^, ^15^, ^16^, ^17^, ^18^ but most reports combine disparate cancers^7^, ^8^, ^9^, ^11^, ^12^, ^14^, ^15^ or those considering lung cancer alone focus on metastasis detection in a single organ, typically bone.^10^, ^13^, ^16^, ^17^, ^18^ Primary studies of WB-MRI in lung cancer staging are predominantly small, single site, explanatory studies with WB-MRI interpretation by a few highly experienced radiologists, which is unlike real-world pathways.^4^ Studies usually compare single modalities (eg, WB-MRI *vs* PET-CT) instead of the multiple staging tests encountered in daily practice.^4^ There are no data regarding how WB-MRI pathways influence staging times, additional tests, costs, or treatment decisions. As such, there is insufficient evidence to assess whether WB-MRI should be adopted.^19^

We did two parallel prospective multicentre trials to elucidate and directly compare the diagnostic accuracy and efficiency of WB-MRI-based staging pathways with standard staging in NSCLC (Streamline L) and colon cancer (Streamline C).^20^ Here, we report findings from Streamline L.

## Methods

### Study design and participants

Streamline L is a multicentre, prospective trial comparing diagnostic accuracy for metastatic disease of staging pathways based on initial WB-MRI, with standard staging in NSCLC. Ethics committee approval was granted on Oct 3, 2012, and the trial was coordinated by Cancer Research UK and University College London Cancer Trials Centre, with oversight from an independent data monitoring committee and a trial steering committee. All patients gave written informed consent.

Patients were recruited from 16 general and teaching UK National Health Service (NHS) hospitals. Because 11 of the 16 sites did not have the infrastructure to do WB-MRI, these sites sent patients to a nearby hospital for scanning (appendix p 2). Eligible patients were aged 18 years or older with histologically proven or suspected NSCLC on chest CT, referred for staging. Suspicion of NSCLC was defined as an abnormality with CT characteristics sufficiently suggestive of NSCLC to indicate additional diagnostic and staging investigations. The disease had to be potentially radically treatable on the diagnostic CT chest, defined as stage IIIb or less (ie, T1–4, N0–2, and M0 by TNM 7^21^). Patients were ineligible if further workup was considered inappropriate by the clinical care team or patient. Histologies other than non-small-cell were ultimately excluded, but patients undergoing treatment based on clinically diagnosed NSCLC remained eligible. Patients were ineligible if they could not provide informed consent, had severe systemic disease making it undesirable to participate, were pregnant, or had contraindications to MRI.

Participants were identified from outpatient clinics, multidisciplinary team meetings, and inpatient wards by local research team, who took informed consent from consecutive, unselected, eligible patients. A screening log detailed all patients approached and reasons for non-participation, where applicable. Age, performance status, sex, and request date for the first staging investigation were collected from recruited patients. Staging completion date was also recorded, defined as the date of the final test in the standard staging pathway.

The protocol has been published^4^ and is available online.

### Procedures

Participants had contemporaneous WB-MRI plus all standard staging investigations done as part of usual clinical care. Standard investigations were generally undertaken at the recruitment site, or a secondary hospital by referral in the case of specialised tests (such as PET-CT), and were interpreted by local consultant radiologists as per usual clinical practice. Interpretation of standard investigations was masked to WB-MRI images and findings. Case report forms included the nature and date of all standard investigations actually done before the first major treatment decision, and their findings regarding presence and location of metastatic disease.

The platform used for WB-MRI was in line with usual practice. A minimum dataset of sequences was acquired, including diffusion, T2-weighted, and T1-weighted (pre-intravenous and post-intravenous gadolinium contrast medium) imaging (appendix p 3). WB-MRI datasets were uploaded electronically to a secure central imaging server (3Dnet; Biotronics3D, London, UK) for interpretation, and were withheld initially from the local Picture Archiving and Communications System to ensure local radiologists interpreting standard staging interventions were masked.

Across all recruitment sites and imaging hubs, 16 radiologists interpreted WB-MRI and were unaware of all other standard staging investigations and clinical information (other than the suspected cancer diagnosis and its lobar location). All radiologists were fellows of the Royal College of Radiologists and had interpreted at least 20 validated staging WB-MRIs. Radiologists with experience of fewer than 100 WB-MRI datasets initially had their reports validated by more experienced colleagues (ie, had worked on >100 WB-MRI datasets) and reported alone only once deemed competent by their colleague. This procedure was designed specifically to mirror how WB-MRI would be reported in NHS practice if more widely disseminated. Radiologists completed case report forms documenting the T and N stage of the local tumour,^21^ and the presence, location, and diameter of metastatic disease across various anatomical sites using six numerical confidence levels grouped subsequently into normal, equivocal, and abnormal. Radiologists interpreted WB-MRI as per their usual practice, considering known morphology and characteristics of metastatic disease across the various MRI sequences,^22^ and reproduced case report form findings in a free text clinical report, uploaded onto the 3Dnet software for subsequent release to the multidisciplinary team meeting. If additional tests were recommended for equivocal findings, this suggestion was included in their report.

Patients were discussed in the multi-disciplinary team meeting at their local hospital as per usual care pathways. WB-MRI images and reports were withheld until patients had completed all standard staging investigations so that the multidisciplinary team made its first major treatment decision based only on standard staging.^4^ The decision was documented (appendix p 4), along with the TNM stage assigned.

In the same meeting, the WB-MRI report and images were then shown to the multidisciplinary team via 3Dnet. The team considered the report and images and stated whether additional tests would have been requested before the first major treatment decision could be reached, had WB-MRI been the initial staging investigation (eg, to investigate equivocal findings). Any such tests were then done if they or an equivalent test had not already been done as part of the standard pathway and the multidisciplinary team considered them essential to patient care. If done already, their results were noted. The multidisciplinary team recorded the TNM stage based on the WB-MRI staging pathway (ie, WB-MRI plus the results of any additional tests generated, if any) and stated what the first major treatment decision would have been on the basis of this pathway. The final multidisciplinary team treatment decision was then made based on all available tests (ie, standard pathway, WB-MRI, and any additional tests; appendix p 4).

We devised a reference standard using multidisciplinary consensus panel review, a procedure that is standard for diagnostic test accuracy studies where an independent reference standard does not exist or is impossible because of incorporation bias.^4^, ^23^ Patients were followed-up for 12 months (or until death, if sooner). Each recruitment site convened a series of panels to derive the reference standard TNM stage, consisting of at least two radiologists (one external to the site) with expertise in cross-sectional imaging and nuclear medicine, and at least one of the following: respiratory physician, thoracic surgeon, or oncologist. The panel had access to a histopathologist if required, and a member of the Cancer Research UK and University College London Cancer Trials Centre and trial management group attended to ensure the consensus process was uniform across the trial. The panel considered all available clinical data over the follow-up period, including images and results of all staging and follow-up investigations, surgical findings, histopathology (surgical resections and biopsies), and patients' clinical course, and assigned a TNM stage for the time of recruitment. The location and size of any metastatic deposits were recorded. In the absence of histological proof, metastatic disease was assumed if new lesions appeared during follow-up with suggestive imaging characteristics, or if compatible lesions that were already present either progressed or responded to therapy. Specific criteria were applied depending on length of follow up (in the case of death) and if the primary tumour remained in situ (appendix p 5). From all follow-up data, the panel assigned a retrospective optimal primary treatment decision, noting radiological perceptual errors in the initial interpretation of staging investigations (ie, unreported metastases that could be identified by the panel in retrospect, with full knowledge of all follow-up investigations).

### Outcomes

The primary outcome was the difference in per-patient sensitivity for metastatic disease detection between standard and WB-MRI staging pathways, compared against the consensus reference standard. Prespecified outcomes were reported according to the diameter of the largest metastatic deposit (≥1 cm or <1 cm) to assess the effect of lesion size on diagnostic accuracy, per-organ sensitivity, and for WB-MRI as a stand-alone investigation based on the original radiologist report.

Secondary outcomes were difference in per-patient specificity for metastatic disease detection between standard and WB-MRI staging pathways, agreement between treatment decisions based on alternate pathways and the multidisciplinary team and consensus panel treatment decisions, staging efficiency (time taken, test number, and costs), per-organ sensitivity and specificity for metastasis, and per-patient agreement for local T and N stage. Additional secondary outcomes related to the effect of differing combinations of MRI sequences on accuracy, interobserver variability in WB-MRI interpretation, and the effect of adding WB-MRI to standard pathways will be reported elsewhere. The comparative patient experience of staging pathways and the findings of a discrete choice experiment have already been reported.^24^, ^25^, ^26^

### Statistical analysis

Using methods for comparative studies,^27^ we estimated that 50 patients with metastasis occult on diagnostic CT chest would give 80% power to detect a sensitivity difference of 24% between WB-MRI (79%) and standard pathways (55%), assuming 25% metastatic prevalence, 53% concordance between pathways, and a 20% withdrawal rate at 1 year, giving a target sample size of 250 patients. On Dec 7, 2015, as recommended by the independent data monitoring committee, the target sample size was revised to 353 patients to ensure inclusion of about 50 patients with metastasis.

We report our prespecified primary and secondary outcomes, and additional sensitivity analyses. Binary comparisons (sensitivity, specificity, and treatment decision agreement) were calculated using paired proportions (population marginal) in STATA 14.2 (College Station, TX, USA). For the primary outcome, equivocal disease was considered positive for metastasis. Sensitivity analysis treated equivocal results as negative.

There were no missing data for the primary outcome. Statistical significance was determined on the basis of 95% CIs from Newcombe paired proportion method;^28^ McNemar's test p values are reported. Pathway treatment decisions were grouped for analysis (see appendix p 6) and compared to the final decisions made by the multidisciplinary team and consensus panel (as a sensitivity analysis). Time to complete staging pathways (excluding initial diagnostic tests) was calculated in days, by adding times for staging tests (from request to performance) to median wait times for a treatment decision by the multidisciplinary team, calculated across all patients. In the case of missing data, median times from the same or similar tests were used. The median difference in time and number of staging tests between pathways was compared for each patient with 95% CI from 2·5 and 97·5 centiles of 1999 bootstrap samples, with replacement used to compare between standard and WB-MRI staging pathways. Descriptive analysis of time to complete staging are reported in median days with IQR for staging pathways.

We compared the costs of WB-MRI versus standard pathways (appendix p 7). The cost analysis was based on a UK NHS perspective. Costs were calculated in pounds sterling (as of 2016–17) and were inflated as necessary. The time horizon was the time from initial diagnosis to treatment decision by the multidisciplinary team. Given the time horizon, which was less than 1 year, discounting was not applied. We calculated the mean cost per patient of tests received when undergoing standard imaging pathways only and WB-MRI (including additional staging tests ordered after the WB-MRI). We only included the cost of the tests received; the costs of the multidisciplinary team were not included because this cost was incurred irrespective of the type of staging test received. We did not include any adverse events related to imaging because no such events were reported. Unit costs were taken from 2016–17 NHS reference costs.^29^ Decisions about which reference costs to use were made with appropriate clinical input (appendix pp 8–9). Mean per-patient staging costs for standard pathways and WB-MRI were compared using 95% CI derived from 1000 bootstrapped replications of the mean with replacement.

Streamline L is registered with the International Standard Randomised Controlled Trial registry, number ISRCTN50436483.

### Role of the funding source

The funder of the study stipulated that the study design should be a diagnostic accuracy trial using a cohort design, but was not involved in data collection, data analysis, data interpretation, or writing of the report. The corresponding author had full access to all the data in the study and had final responsibility for the decision to submit for publication.

## Results

**Figure 1:**
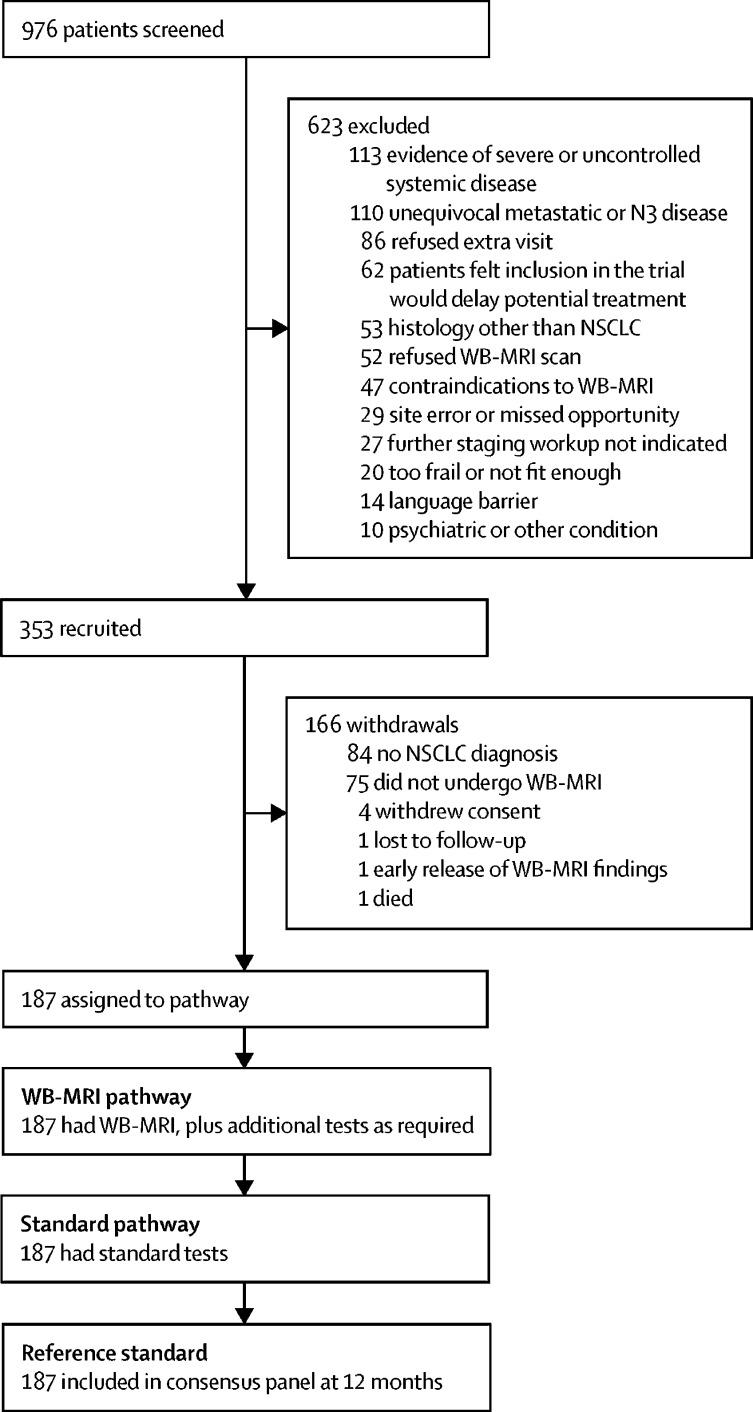
Trial profile NSCLC=non-small-cell lung cancer. WB-MRI=whole-body MRI.

Between Feb 26, 2013, and Sept 5, 2016, 976 patients were screened for eligibility (figure 1). 353 patients were recruited, of whom 166 were excluded, mainly owing to a final diagnosis other than lung cancer (figure 1). The final cohort of 187 patients had a median age of 67 years (IQR 61–75) and 70 (37%) were women (figure 1, table 1). According to the consensus reference standard, 137 (73%) patients were stage T2 or above, 77 (41%) were node-positive (appendix p 10), and 52 (28%) had metastatic disease at the time of staging (appendix p 11), meeting sample size stipulations. In eight patients with metastatic disease at the time of staging (according to protocol definitions, appendix p 5), metastasis only became apparent during follow-up and was not visible on initial staging investigations, even in retrospect.

**Table 1:** Baseline characteristics of final trial cohort

		**Value**
**Sex**		
Male		117 (63%)
Female		70 (37%)
**Age, years**		
Median (IQR)		67 (61–75)
Range		37–96
**Performance status**		
Fully active		86 (46%)
Ambulatory		
	Able to work	75 (40%)
	Not able to work	8 (4%)
Not recorded		18 (10%)
Tumour location*		
Right upper lobe		73 (39%)
Right middle lobe		14 (7%)
Right lower lobe		24 (13%)
Left upper lobe†		54 (29%)
Left lower lobe		28 (15%)
**Histological subtype**		
Adenocarcinoma		115 (62%)
Large cell		4 (2%)
Squamous		42 (22%)
Adenosquamous		1 (1%)
Other		13 (7%)
No histology or missing		12 (6%)

Data are n (%) unless otherwise stated.

* By consensus reference standard. Some patients have multiple tumour locations.

† Including the lingula.

**Figure 2:**
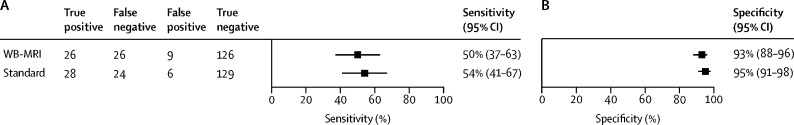
WB-MRI and standard staging pathways sensitivity and specificity for patients with metastatic disease against the consensus reference standard WB-MRI=whole-body MRI.

Sensitivity of staging for patients with metastatic disease was 50% (95% CI 37–63) for WB-MRI and 54% (41–67) for standard pathways, a difference of 4% (−7 to 15, p=0·73; figure 2, table 2). For the primary outcome, there were seven perceptual errors in the WB-MRI pathway and three in the standard pathway. No adverse events (serious or non-serious) were reported during the trial.

**Table 2:** Per-patient sensitivity and specificity for metastatic disease

	Patients with metastatic disease*	**Sensitivity**				Patients without metastatic disease*	**Specificity**			
		WB-MRI staging pathway†	Standard staging pathway	Difference	p value		WB-MRI staging pathway†	Standard staging pathway	Difference	p value
Diagnostic accuracy	52	50% (37 to 63)	54% (41 to 67)	−4% (−15 to 7)	p=0·73	135	93% (88 to 96)	95% (91 to 98)	−2% (−7 to 2)	p=0·45
Equivocal lesions considered negative	52	48% (35 to 61)	46% (33 to 59)	2% (−11 to 14)	..	135	94% (89 to 97)	97% (93 to 99)	−3% (−6 to 1)	..

Data are n or % (95% CI).

* Patients by consensus reference standard.

† WB-MRI plus additional generated tests.

Specificity did not differ between the WB-MRI pathway (93% [88–96]) and standard pathway (95% [91–98], p=0·45). The number of equivocal results per pathway is shown in the appendix (p 12). Sensitivity analysis found no differences between pathways when lesions reported as equivocal were treated as either all positive or all negative (table 2), or across individual organ sites (appendix p 13). The WB-MRI pathway had 82% (64–92) sensitivity for patients whose largest metastasis was at least 1 cm, which did not differ from standard pathways (75% [57–87]); for those with metastasis smaller than 1 cm, sensitivity was 9% (3–28; appendix p 14). As a stand-alone investigation (ie, without additional tests generated), WB-MRI had a similar sensitivity to that of the standard pathway, but had lower specificity than the standard pathway (appendix p 15).

The WB-MRI pathway had 65% agreement for N stage compared with 75% for the standard pathway, a significant difference of 10% (3–18; appendix p 16). Of the 187 patients, 109 had histological proof of N stage, usually via endobronchial ultrasound nodal sampling or surgery, or both. In these patients, there remained a difference in agreement of 10% (1–19) between WB-MRI and standard pathways (appendix p 17). Pathways did not significantly differ in terms of agreement for T stage (appendix p 18).

Agreement with the final treatment decision of the multidisciplinary team was 98% for WB-MRI and 99% for the standard pathway (table 3). Treatment decisions based on WB-MRI and standard pathways had similar levels of agreement with the retrospective consensus panel optimal treatment decision (appendix p 19).

**Table 3:** Agreement between pathway and multidisciplinary team treatment decisions

	n*	**WB-MRI staging pathway**†		**Standard staging pathway**		**Difference agreement, % (95% CI)**
		Agreement	Disagreement	Agreement	Disagreement	
All patients	183	180 (98%)	3 (2%)	181 (99%)	2 (1%)	−1% (−4 to 2)
Patients with metastatic disease	52	51 (98%)	1 (2%)	50 (96%)	2 (4%)	2% (−7 to 11)
Patients without metastatic disease	131	129 (98%)	2 (2%)	131 (100%)	0	−2% (−4 to 1)

Data are n (%) unless otherwise stated.

* Four patients were missing at least one type of patient treatment decision.

† WB-MRI plus additional generated tests.

Across the cohort, standard staging pathways involved 302 individual investigations and WB-MRI involved 232 individual investigations; WB-MRI pathways generated an additional 45 tests (appendix pp 20–21). The median number of tests did not differ between the WB-MRI (one [1 to 1]) and standard (one [1 to 2]) pathways (difference 0 [–1 to 0]; appendix p 22).

**Figure 3:**
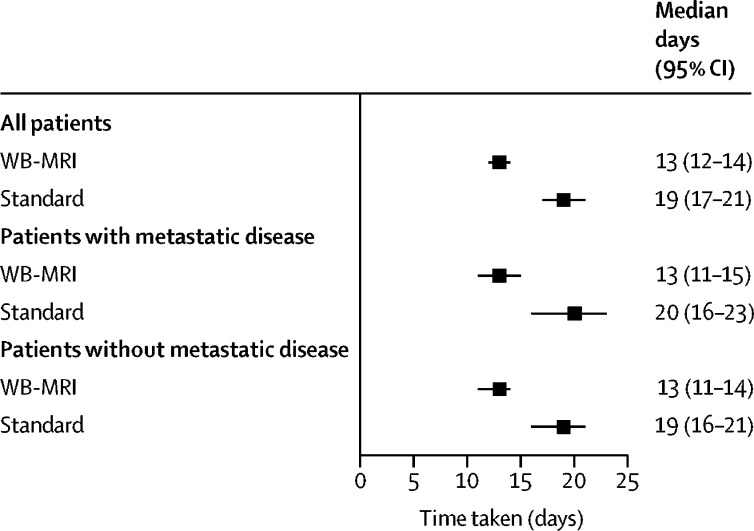
Time taken for staging pathways WB-MRI=whole-body MRI.

Time to staging was shorter for WB-MRI pathways than for standard pathways (13 days [12–14] *vs* 19 days [17–21]); a difference of 6 days (4–8) (figure 3, appendix pp 23–24). Mean per-patient costs for the WB MRI pathway (£317 [273–361]) were lower than for the standard staging pathway (£620 [574–666]; appendix p 25).

## Discussion

To date, Streamline L is the largest prospective multicentre trial to compare the diagnostic accuracy of WB-MRI and standard staging pathways for metastatic disease in patients with newly diagnosed NSCLC. Both pathways showed similar accuracy, but the WB-MRI pathway was more time-efficient and cost-efficient. Treatment decisions were similar. Our data suggest WB-MRI is a viable replacement for standard pathways.

WB-MRI pathways had no advantage over standard pathways in terms of diagnostic accuracy. The overall sensitivity of both pathways for metastatic disease was lower than published studies^10^, ^13^ suggest, although 2018 data^30^ challenges the accuracy of standard staging pathways. We excluded patients with locally advanced or metastatic disease on their diagnostic CT chest (including the lower neck, liver, and adrenal glands) because these patients generally undergo treatment without curative intent. Such exclusion is unusual in the literature. Metastases were therefore either occult or involved remote sites. Eight patients developed their first metastasis during follow-up which were not visible in retrospect on any staging examination. The concept of occult metastatic disease is well established: 35% of patients develop metastatic disease post thoracotomy despite a negative staging PET-CT.^31^ The number of perceptual errors was low, and many retrospectively visible lesions were subtle and difficult to detect prospectively. As a pragmatic trial, Streamline L provides the best estimate of NSCLC staging accuracy in routine clinical practice.

We found that the WB-MRI pathway had 82% sensitivity for patients with metastatic disease of at least 1 cm, compared with only 9% for smaller metastasis. Our WB-MRI protocol complied with accepted international standards,^32^ including diffusion weighted imaging and post-gadolinium sequences; however, by necessity, had to compromise—for example, on slice thickness—to ensure reasonable total scan times. The previous largest study of WB-MRI was a single site comparison with PET-CT alone,^22^ which reported WB-MRI had a per-patient sensitivity of 70% and specificity of 92%, compared with 63% and 95% for PET CT, respectively. However, unlike Streamline L, imaging interpretation was done via the consensus of two experienced readers, and complete staging pathways were not evaluated. The effect on treatment decisions was not considered.

We found WB-MRI pathways had similar accuracy for T staging compared with standard pathways, suggesting the anatomical information given by WB-MRI matched that of standard imaging. Sensitivities for N stage were comparable to that previously reported,^17^, ^18^ but standard pathways were superior overall and in those with histological proof of N stage. It is widely accepted that invasive nodal staging with endobronchial ultrasound (and EUS where available) is superior to imaging techniques for detecting nodal metastases^33^ and current guidelines recommend sampling of enlarged mediastinal nodes if it would affect patient management.^34^ We specifically investigated implementation of WB-MRI after diagnostic CT, which was therefore also available for lymph node size measurement as part of the clinical decision making for this pathway. The 2019 NICE guidelines^35^ recommend a systematic approach to staging hilar and mediastinal nodes with increased use of endobronchial ultrasound-guided sampling. Endobronchial ultrasound was available to all Streamline L recruitment sites as part of patient diagnostic and staging workup. The lower sensitivity of WB-MRI for nodal staging will likely be offset by the current invasive approach to N staging if status affects treatment decisions. Furthermore, nodal stage alone does not dictate treatment; for example, patients staged N0, N1, and, in some cases, N2 disease are still candidates for surgery, and patients with metastatic disease are treated accordingly, regardless of nodal stage. In support, the lower sensitivity of the WB-MRI pathway did not negatively affect treatment decisions. Agreement with both the final multidisciplinary team treatment decision and the optimal retrospective treatment decision was similar for both staging pathways, suggesting that WB-MRI could replace standard pathways without patient detriment.

Generally, efficiency receives less attention than diagnostic accuracy.^19^ Timeliness of lung cancer treatment is a care quality indicator; reducing time to treatment decisions by 2 weeks is associated with improved survival^33^ and prolonged pathways increase patient anxiety.^6^ Streamline L found that WB-MRI pathways were more efficient than standard pathways, reducing the time to complete staging significantly and decreased average per-patient staging costs by £303, largely due to PET-CT use by standard pathways. Efficiency of WB-MRI pathways could potentially increase given the growth of routine cranial imaging in staging, and the emphasis on oligometastatic (M1b) disease detection in the eighth edition of TNM. On average, the generation of additional tests to the WB-MRI pathway added 4–5 days to the pathway staging time. Although MRI access is restricted in many health-care settings, our data suggest that increased provision would ultimately reduce the cost and complexity of staging NSCLC. Although patients report that having WB-MRI is a greater burden than standard imaging,^24^ a discrete choice experiment^26^ done as part of the trial shows patients generally prefer WB-MRI staging to standard pathways if they reduce staging times and radiation exposure as found in Streamline L.

A strength of our trial is its pragmatic design. We recruited from a representative range of general and teaching hospitals, with all imaging done and interpreted according to usual local protocols, to increase generalisability of our results. The 16 radiologists interpreting WB-MRI were representative of those who would do so in daily NHS practice. We avoided using a smaller number of highly experienced radiologists; although we acknowledge that such individuals might achieve sensitivities greater than we report, they do not represent the national workforce. We used multidisciplinary team meetings to mirror patient care in the NHS. In doing so, we captured the entirety of standard pathways, including contemporaneous treatment decisions. We used a novel cloud-based image repository to maintain blinding and control multidisciplinary team access to WB-MRI until the appropriate time in the decision-making process. We were able to model the content and timing of WB-MRI staging pathways, and the potential effect on decision making. Conversely, previous research usually reports head-to-head comparisons between single imaging platforms, failing to capture pathway complexity. To our knowledge, our trial design is unique.

Streamline L does have limitations. Our withdrawal rate was superficially high, but most excluded participants were excluded because of a final diagnosis other than NSCLC. We masked radiologists reporting WB-MRI to patient history and contemporaneous imaging. This was masked to isolate diagnostic test accuracy within a pragmatic setting. Participants were representative of those undergoing staging in daily practice, although we did exclude pregnant women, patients not wanting to undergo WB-MRI, and patients with contraindications to MRI. We modelled timing of WB-MRI staging pathways on the basis of real waiting times collated from recruitment sites during the trial, although sites had capacity to do WB-MRI. Waiting times might not be representative of those at other hospitals, and in other countries. Some of the benefits of reduced staging time by WB-MRI pathways could be negated if time to commencing treatment (eg, surgical resection) are not reduced in parallel. Treatment decisions based on WB-MRI pathways were made after the multidisciplinary team was unmasked to all standard imaging tests, which could introduce bias. However, this situation was unavoidable if the full complexity of standard staging pathways was to be captured without interference from WB-MRI findings and if treatment decisions were to be recorded contemporaneously. Furthermore, alternate pathway agreement with a retrospective optimal treatment at 12 months remained very similar. Our cost analyses reflect an English NHS perspective and could differ in other settings, which might negate some of the cost advantages of WB-MRI pathways. Although WB-MRI is advocated as being safer than current standard staging investigations, new technologies are reducing radiation dose,^36^ and there are current uncertainties about the neuronal deposition of gadolinium.^37^ Further research is needed to define the potential use of WB-MRI in the assessment of treatment response and post-therapy surveillance for recurrent disease. Our findings are specific for NSCLC and might not be relevant to other primary tumour sites.

In summary, WB-MRI staging pathways have similar diagnostic accuracy to standard pathways for identifying patients with metastatic disease in newly diagnosed NSCLC, and lead to similar treatment decisions. However, they reduce staging time and costs. In a real-world NHS setting, WB-MRI-based pathways are a viable replacement for standard pathways.

## Data sharing

Individual participant data that underlie the results reported in this article, after deidentification (text, tables, figures, and appendices), will be available for individual participant data meta-analysis beginning 9 months and ending 36 months after article publication. Data will be available to investigators whose proposed use of the data has been approved by an independent review committee (learned intermediary) identified for this purpose. Data access requires proof of relevant ethical committee approval for the specified analysis only. Data will be limited to those required for a specific analysis to protect deanonymisation. Where proposals that would compete with ongoing or planned research from the investigators within the trials team, data access will only be granted once investigator team publications are submitted. Proposals should be directed to the corresponding author; to gain access, data requestors will need to sign a data access agreement. After 36 months, there is no funded technical support. Information regarding submitting proposals and accessing data can be obtained by emailing ctc.enquiries@ucl.ac.uk
