# Supplementary material for: Diagnostic accuracy of whole-body MRI versus standard imaging pathways for metastatic disease in newly diagnosed non-small-cell lung cancer: the prospective Streamline L trial
Source: Lancet Respir Med. 2019 Jun;7(6):523–32. doi: 10.1016/S2213-2600(19)30090-6 (PMC6529610; doi:10.1016/S2213-2600(19)30090-6)
Supplement: Supplementary appendix [file mmc1.pdf]

THE LANCET
Respiratory Medicine

Supplementary appendix

This appendix formed part of the original submission and has been peer reviewed. We post it as supplied by the authors.

Supplement to: Taylor SA, Mallett S, Ball S, et al. Diagnostic accuracy of whole-body MRI versus standard imaging pathways for metastatic disease in newly diagnosed non-small-cell lung cancer: the prospective Streamline L trial. *Lancet Respir Med* 2019; published online May 9. http://dx.doi.org/10.1016/xS2213-2600(19)30090-6.

Online appendix

Contents

Streamline Investigators 1
Recruitment sites and Imaging hubs 2
WB-MRI protocol minimum dataset 3
Summary of information recorded by the MDT and categories of treatment decisions 4
Criteria for diagnosis of metastatic disease by the consensus reference panel 5
Grouping of treatment decision for analysis 6
Justification for economic analysis 7
Streamline L Test Unit costs from NHS Reference Costs 2016/17 8
Appendix Table 1:T and N stage of the final trial cohort based on consensus reference standard 10
Appendix Table 2:Sites of metastatic disease according to the consensus reference standard 11
Appendix Table 3:Equivocal results for metastatic disease 12
Appendix Table 4:Per organ sensitivity and specificity for metastatic disease- WB-MRI staging pathways versus standard staging pathways, against the consensus reference standard 13
Appendix Table 5:Per patient sensitivity and specificity for metastatic disease according to the size of the largest deposit- WB-MRI staging pathways versus standard staging pathways, against the consensus reference standard 14
Appendix table 6:Per patient sensitivity and specificity for metastatic disease- WB-MRI as a standalone investigation versus standard staging pathway, against the consensus reference standard 15
Appendix Table 7:Per patient agreement for N stage- WB-MRI staging pathways versus standard staging pathways, against the consensus reference standard 16
Appendix Table 8:Per patient agreement for tumour N stage-standard staging pathways versus WB-MRI staging pathways against the consensus reference standard in patients with histological proof of N stage 17
Appendix Table 9:Per patient agreement for tumour T stage- WB-MRI staging pathways versus standard staging pathways, against the consensus reference standard 18
Appendix Table 10:Agreement between treatment decisions based on the WB-MRI and standard staging pathways and the retrospective 12-month consensus panel optimal treatment decision 19
Appendix Table 11:Investigations performed as part of the standard staging pathway 20
Appendix Table 12:Additional Investigations generated by WB-MRI 21
Appendix Table 13:Number of tests required to complete staging according to the staging pathway 22
Appendix Table 14:Time to complete staging according to staging pathway (95% CI) 23
Appendix Table 15:Time to complete staging according to staging pathway-interquartile range 24
Appendix Table 16:Mean per patent staging cost according to staging pathway 25

Streamline Investigators

Ruth Evans, Revanth Jannapureddy, Tina Mills-Baldock, Kishor Barhate, Zoltan Nagy, Sherif Raouf, Akosa Aboagye, Girija Anand, Rommel Butawan, Elizabeth Hadley, Adesewa Onajobi, Tanjil Nawaz, Catherine Norman, Nathalie Rich, Khawaja Shahabuddin, Sidra Tulmuntaha, Shafi Ahmed, Louise Lim, Fiona McKirdy, Jenna Couture, Shahanara Ferdous, Payal Julka, Ali Mohammed, William Ricketts, Mohamed A Thaha, Marie Jackson, Clive Kay, Andy Lowe, Janet McGowan, Amjad Mohammed, Jon Robinson, Lara Curry, Sasithar Maheswaran, Subramanian Ramesh, Pippa Riddle, Shaki Balogun, Yvonne Campbell, Nelesh Jeyadevan, Aji Kavidasan, Imogen Locke, Tuck-Kay Loke, Ibiyemi Olaleye, Clare Collins, Elizabeth Green, Colm Prendergast, Thida Win, Amy Davis, Lyn Blakeway, Sofia Gourtsoyianni, Adrian Green, Christian Kelly-Morland, Sahar Naaseri, David Snell, Dorothee Boisfer, Keyury Desai, Balinder Hans, Sophia Hans, Eleni Ntala, Adnam Alam, Stephen Burke, Helen Pardoe, Sanjaya Wijeyekoon, Nishat Bharwani, Gule Hanid, Lesley Honeyfield, Tina Stoycheva, Katherine van Ree, Dominic Blunt, Farid Bazari, Helen Beedham, Jane De Los Reyes Lauigan, Priya Limbu, Nicola Lucas, Sally O'Connor, Anita Rhodes, Laletha Agoramoorthy, Martha Handousa, Abel Jalloh, Stefania Stegner, Shanna Wilson, David Birch, Suzanne Chukundah, John O'Donohue, Priscilla Phiri, Raj Srirajaskanthan, Eleni Karapanagiotou, Daniel Smith, Ferrial Syeed, Chloe van Someren, Rudi Borgstein, Jamila Roehrig, David Chao, Lorraine Hurl, Andrew Gogbashian, Andre Nunes, Ian Simcock, James Stirling, Richard Beable, Maureen Furneaux, Nicola Gibbons, Antony Higginson, Howard Curtis, Kitrick Perry, Anita Amadi, Heather Hughes, Prital Patel, Matthew Train, Gary Atkin, Colin Elton, Stephen Karp, Lisa Woodrow, Dominic Yu, Sajid Khan, Alistair Rienhardt, Pooja Datt, Rajapandian Ilangovan, Ian Jenkins, Saba Mahmud, Uday Patel, Michael Long, Teresa Light, Joanne Kellaway, Ann O'Callaghan, William Partridge, Amelia Daniel, Ugo Ekeowa, Erica Scurr, Veronica Morgan, Nina Tunariu, Elizabeth Chang, Laura Hughes, Ellice Marwood, Katie Prior, Meena Reddi, Kara Sargus, Abby Sharp, Teresita Beeston, Elizabeth Isaac, Adoracion Jayme, Jagadish Kalasthry, Wivijin Piga, Farzana Rahman, Shraddha Weir, Aileen Austria, James Crosbie, Alec Engledow, Jonathan McCullogh, Austen Obichere, Kai-Keen Shiu, Christopher Wanstall, Celia Simeon, Amy Smith, Andrew Bateman, David Breen, Liane Davis, Chris Everitt, Alice Johnson, Paul Nichols, Beth Shepherd, Kayleigh Gilbert, Azmina Verjee, Michelle Saull, Jonathan Wilson, Rashidat Adeniba, Veronica Conteh, Sarah Howling

Recruitment sites and Imaging hubs

Recruitment site	Imaging hub
University College Hospital London	University College Hospital London
Barnet Chase Farm Hospitals	
Princess Alexandra Hospital, Harlow	
North Middlesex	
Whittington Hospital	
Queen’s Hospital. Romford	
Lister Hospital, Stevenage	Paul Strickland Scanner Centre, Mount Vernon
Croydon University Hospital	Royal Marsden Hospital
Kingston Hospital	
Charing Cross Hospital	Charing Cross Hospital
Homerton Hospital[a]	Homerton Hospital
Guy’s and St Thomas Hospital	Guy’s and St Thomas Hospital
Lewisham Hospital	
St Bartholomews Hospital	St Bartholomews Hospital/Royal London Hospital
Whipps Cross Hospital	
Newham Hospital	

[a]1 patient underwent WB-MRI at St Bartholomew’s Hospital and 1 at University College London Hospital

WB-MRI protocol minimum dataset
Scanning maybe performed at either 1.5T or 3T.
Whole body is head to mid-thigh

1. Whole-body diffusion weighted imaging:

Axial: STIR-EPI (or other fat sat technique) diffusion weighted imaging. Fixed slice thickness of 5mm to 7mm (to match T2 and T1 weighted axials as below) two b-values (b50 and b900). A minimum acquisition matrix of 128 x 128 (or an interpolated equivalent) (rectangular FOV should be used if available and appropriate for the patient), as a reference a minimum SNR of 6 on b50 images (for liver) should be maintained by increasing the number of averages. All imaging should be performed in gentle respiration (recommended as 4 stations of 50 slices beginning from the vertex to mid thighs). Diffusion imaging through the brain is optional.

2. Whole-body T2 weighted imaging:

Axial*:* Axial T2 weighted (without fat-suppression) imaging, maximum 5 to 7 mm slice thickness. Where possible, respiratory and ECG triggering should be used for the chest, respiratory triggering alone for the upper abdomen.

Pre-contrast T1 weighted imaging:

DIXON Technique to be applied if available.

a. **Axial**: Whole-body T1 GRE (e.g. Flash 2D) non-contrast enhanced non fat sat. Image resolution and slice thickness should be ideally matched to T2 weighted imaging.

OR

b. **Coronal**: T1 fat saturated volume interpolated gradient echo imaging (e.g. 3D) pre contrast.

Post-contrast T1 weighted imaging (if gadolinium not contraindicated or refused):

Minimum data set
Axial liver (60-70 sec)
Axial lung (equilibrium phase)
SFOV axial head
Optional
Coronal (organ specific or whole body)

a. **Axial:** post contrast e.g. T1 fat saturated volume interpolated gradient echo imaging (3D) breath hold of the *liver (60-70 seconds delay) and lungs*. Multiple breath-holds employed to provide full volume coverage if required. A minimum of a 256x256 (rectangular FOV acquisition if possible and appropriate for the patient) acquisition matrix should be employed. 5-7 mm slice thickness.

b. **Coronal:** post contrast whole body; e.g. T1 fat saturated volume interpolated gradient echo imaging (3D) and post contrast. Slice thickness 5mm. Breath Hold.

c. **Axial:** fat saturated T1 weighted imaging of the brain (SFOV). An acquisition matrix of 256 x 256 should be employed

Summary of information recorded by the MDT and categories of treatment decisions

- Stage and treatment decision based on standard investigations (and the number, timing, nature and findings of these investigations).
- Stage and theoretical treatment decision based on WB-MRI staging pathway (and the number, timing, nature and findings of additional tests generated, if any).
- Final treatment decision incorporating all available tests.

Categories of treatment decisions

- Surgical removal of primary
- Radical Radiotherapy
- Chemotherapy
- Radiotherapy (non radical)
- Combination chemo-radiotherapy
- Supportive/Palliative care
- Other-describe

Criteria for diagnosis of metastatic disease by the consensus reference panel

- For patients in whom the primary tumour was completely removed within 3 months of diagnosis, all new metastatic sites identified over the follow up period were be assumed to have been present at diagnosis
- If the primary tumour was left in situ for more than 3 months of diagnosis (or there was incomplete removal), new metastatic sites were assumed to have been present at diagnosis if they were identified within 6 months of diagnosis   If new metastatic sites were diagnosed beyond 6 months of diagnosis, and there was no evidence of their presence on retrospective review of all staging investigations, they were assumed to be new disease and not present at diagnosis
- If patients with tumours left in situ did not undergo any imaging capable of detecting metastatic disease within 6 months of diagnosis of the primary and new metastatic sites were apparent beyond 6 months but not visible in retrospect on any trial imaging, the consensus panel decided if the disease was likely present at diagnosis, based on its location, size and imaging characteristics.
- If a patient died before the 12 months follow up, the panel reviewed all available imaging, histopathology and clinical course prior to death and in consensus decided if a confident diagnosis of the presence or absence of metastatic disease could be made (for example the presence of imaging characteristics compatible with metastasis and no alternative explanation, or if lesions with characteristics compatible with metastasis that either grew or shrunk (on therapy). If this judgement could not be made with confidence (for example if the patient had equivocal lesions on staging investigations and no further follow up), patients were not be excluded but multiple imputation used to account for missing data

Grouping of treatment decision for analysis

Treatment decision category	Treatment decisions included
Curative intent	• Surgical removal of primary<br>• Radical Radiotherapy<br>• Combination chemo-radiotherapy
Non-curative intent	• Chemotherapy<br>• Radiotherapy (non radical)<br>• Supportive/Palliative care

Justification for economic analysis

As per the trial protocol, a full economic evaluation was not performed because of the observed concordance between WB-MRI and conventional staging tests in informing treatment decisions. The care pathway may be divided into two stages: the treatment decision pathway and the subsequent disease pathway. The former includes the time from initial diagnosis to treatment decision by the MDT; the latter includes the time period following the treatment decision. If there is no difference in treatment decisions made with the two different staging methods, the only difference in costs assigned to the two staging methods is the differential costs of the two sets of staging tests (i.e. standard staging vs. WB-MRI pathway, including additional tests requested), and there will be no difference in treatment pathways or outcomes on the basis of the experimental staging tests used and the resulting costs. As specified in the trial protocol, concordance between conventional staging and WB-MRI was defined as the situation in which >90% of treatment decisions were the same using both imaging methods, or <10% treatment decision were different. Discordance was defined as the case in which >10% treatment decisions were different, or <90% were the same. In the protocol we specified that in the case of concordance, the economic analysis would focus on the cost of the treatment decision pathways only, because the disease pathways will be no different. In this case the cost-effectiveness of WB-MRI versus conventional staging algorithms depends only on the incremental cost (positive or negative) of WB-MRI versus conventional staging algorithms in the treatment decision pathway. Conversely, if there is discordance between the treatment decisions, suggesting that patients would have received different treatment depending on which of the two experimental staging methods was used, then the economic analysis ought to include both the treatment decision pathways and the subsequent disease pathways because both of these will vary between WB-MRI and conventional staging algorithms. In this case the cost-effectiveness of WB-MRI depends on the incremental cost of the WB-MRI versus conventional staging algorithms in the treatment decision pathway plus the incremental costs and health benefits of the disease pathway.
The agreement with the MDT final treatment decision was 98% and 99% for WB-MRI and standard pathways respectively, clearly indicating there was concordance between conventional staging and WB-MRI. On this basis, as specified in our trial protocol, the economic analysis focused on a comparison of the costs of the treatment decision pathways only, which is the analysis included in the manuscript.

Streamline L Test Unit costs from NHS Reference Costs 2016/17

**Currency code**	**Currency Description**	**Tests**	**Mean unit cost (£)**
RD03Z	Magnetic Resonance Imaging Scan of One Area, with Pre- and Post-Contrast	MRI adrenals<br>MRI head	180.35
RD01A	Magnetic Resonance Imaging Scan of One Area, without Contrast, 19 years and over	MRI - Other<br>MRI MSK	139.30
RD05Z	Magnetic Resonance Imaging Scan of Two or Three Areas, with Contrast	MRI - WB-MRI	206.51
RN03A	Positron Emission Tomography with Computed Tomography (PET-CT) of more than Three Areas, 19 years and over	PET CT	484.17
RD21A	Computerised Tomography Scan of One Area, with Post-Contrast Only, 19 years and over	CT chest	97.39
YD03Z	Percutaneous Biopsy of Lesion of, Lung or Mediastinum	CT guided biopsy	791.50
RD22Z	Computerised Tomography Scan of One Area, with Pre- and Post-Contrast	CT head	120.07
RD21A	Computerised Tomography Scan of One Area, with Post-Contrast Only, 19 years and over	CT liver	97.39
RD24Z	Computerised Tomography Scan of Two Areas, with Contrast	CT abdomen and pelvis<br>CT chest and abdomen	112.33
RD26Z	Computerised Tomography Scan of Three Areas, with Contrast	CT chest, abdomen and pelvis<br>CT neck, chest and abdomen	122.51
RN15A	Nuclear Bone Scan of Two or Three Phases, 19 years and over	Bone scan	292.40
EBUS	Endobronchial Ultrasound Examination of Mediastinum and Percutaneous Biopsy of Lesion of, Lung or Mediastinum	EBUS and EBUS/TBNA	1441.17
RD40Z	Ultrasound Scan with duration of less than 20 minutes, without Contrast	Ultrasound	51.78
YJ04Z	Core Needle Biopsy of Axillary Lymph Nodes	Ultrasound guided biopsy	92.43
RD97Z	Admission or Attendance for Diagnostic Imaging	X ray	18.71
FE35Z	Diagnostic Flexible Sigmoidoscopy, 19 years and over	Sigmoidoscopy	169.43
RD42Z	Ultrasound Scan with duration of 20 minutes and over, without Contrast	Rectal ultrasound	64.95

DZ70Z	Endobronchial Ultrasound Examination of Mediastinum	EUS	649.67
DZ69A	Diagnostic Bronchoscopy, 19 years and over	Bronchial washing	686.15

Appendix Table 1
T and N stage of the final trial cohort based on consensus reference standard

**T Stage**[a]	**N Stage**[a]			
	N0	N1	N2	N3
T1a	29	1	1	0
T1b	12	2	4	1
T2a	33	7	10	2
T2b	15	2	5	1
T3	10	6	9	8
T4	11	3	7	8

[a]87 patients with histological proof for both T & N stage, 13 patients with histological proof for T stage only and 24 patients with histological proof for N stage only

Appendix Table 2
Sites of metastatic disease according to the consensus reference standard

			Imaging diagnosis if no histological proof [b]		
**Site**	**Number of patients [a]**	**Histological proof n (%)**	**Characteristic imaging appearances n (%)**	**Growth on follow up n (%)**	**Response to therapy n (%)**
Liver	9	2 (22)	6 (86)	4 (57)	1 (14)
Lung	14	0 (0)	14 (100)	11 (79)	3 (21)
Pleura	5	1 (20)	4 (100)	2 (50)	1 (25)
Brain	14	1 (7)	13 (100)	8 (62)	4 (31)
Adrenal	7	0 (0)	6 (86)	5 (71)	1 (14)
Bone	15	1 (7)	14 (100)	7 (50)	2 (14)
Other [c]	9	2 (15)	11 (100)	5 (45)	0 (0)

[a] Patients may have more than one site of metastatic disease
[b] Metastasis may fulfil more than one criterion for imaging diagnosis
[c] Nine patients with metastasis in thirteen other sites (pancreas, soft tissue limbs, para aortic node, soft tissue abdomen/pelvis, right thyroid, left axilla, soft tissue neck/chest)

Appendix Table 3
Equivocal results for metastatic disease

	**Equivocal results (n)**			
	**Patients with metastatic disease[a]**		**Patients without metastatic disease[a]**	
	WB-MRI staging pathway[b]	Standard staging pathway	WB-MRI staging pathway [b]	Standard staging pathway
Per patient	1	4	1	2
Non skeletal sites[c]	3	11	8	17
Skeletal sites[c]	4	4	1	0

[a] Patients by consensus reference standard
[b] WB-MRI plus additional generated tests
[c] Per organ as multiple sites can have equivocal results

Appendix Table 4
Per organ sensitivity and specificity for metastatic disease- WB-MRI staging pathways versus standard staging pathways, against the consensus reference standard

	Sensitivity % (CI 95%)[a]				Specificity % (CI 95%)[a]			
Site	Number with metastatic disease[b]	WB-MRI staging pathway[c]	Standard staging pathway	Difference WB-MRI[c] - Standard	Number without metastatic disease[b]	WB-MRI staging pathway[c]	Standard staging pathway	Difference WB-MRI[c] - Standard
Liver	9	44 (19 to 73)	33 (12 to 65)	11 (-23 to 46)	178	99 (97 to 100)	100 (98 to 100)	-1 (-2 to 1)
Lung	14	36 (16 to 61)	36 (16 to 61)	0 (-22 to 22)	173	97 (94 to 99)	98 (95 to 99)	-1 (-4 to 3)
Pleura	5	80 (38 to 96)	40 (12 to 77)	40 (7 to 73)	182	97 (94 to 99)	98 (95 to 99)	-1 (-4 to 2)
Brain	14	57 (33 to 79)	44 (21 to 67)	13 (-5 to 33)	173	99 (97 to 100)	100 (98 to 100)	-1 (-2 to 1)
Adrenal	7	28 (8 to 64)	57 (25 to 84)	-29 (-71 to 14)	180	98 (95 to 99)	98 (95 to 99)	0 (-3 to 3)
Bone	15	60 (36 to 80)	67 (42 to 85)	-7 (-31 to 18)	172	99 (96 to 100)	99 (96 to 100)	0 (-3 to 3)
Other	9	22 (6 to 55)	44 (19 to 73)	-22 (-56 to 12)	178	97 (94 to 99)	93 (89 to 96)	4 (0 to 8)

[a] equivocal results considered positive
[b] Patients by consensus reference standard
[c] WB-MRI plus additional generated tests

Appendix Table 5

Per patient sensitivity and specificity for metastatic disease according to the size of the largest deposit- WB-MRI staging pathways versus standard staging pathways, against the consensus reference standard

		Sensitivity % (CI 95%)[a]				Specificity % (CI 95%)[a]		
	**Number with metastatic disease[b,c]**	**WB-MRI staging pathway[d]**	**Standard staging pathway**	**Difference (P value)**	**Number without metastatic disease[b,c]**	**WB-MRI staging pathway[d]**	**Standard staging pathway**	**Difference (P value)**
Maximum metastatic deposit size ≥1cm	22	82 (64 to 92)	75 (57 to 87)	7 (-9 to 23)	135	93 (88 to 96)	95 (91 to 98)	-2 (-7 to 2) p=0·45
Maximum metastatic deposit size <1cm	28	9 (3 to 28)	27 (13 to 48)	-18 (-37 to 1)				

[a] equivocal results considered positive
[b]Patients by consensus reference standard
[c]2 patients missing largest metastatic disease size
[d]WB-MRI plus additional generated tests

Appendix table 6
Per patient sensitivity and specificity for metastatic disease- WB-MRI as a standalone investigation versus standard staging pathway, against the consensus reference standard

	**Sensitivity % (CI 95%)[a]**				**Specificity % (CI 95%)[a]**			
	Number with metastatic disease[b]	WB-MRI alone[b]	Standard staging pathway	Difference: WB-MRI[c] - Standard	Number without metastatic disease[b]	WB-MRI alone[c]	Standard staging pathway	Difference: WB-MRI[c] - Standard
Diagnostic accuracy	52	50 (37 to 63)	54 (41 to 67)	-4 (-16 to 8)	135	85 (78 to 90)	95 (91 to 98)	-10 (-17 to -4)

[a] equivocal results considered positive
[b] Patients by consensus reference standard
[c] WB-MRI alone

Appendix Table 7
Per patient agreement for N stage- WB-MRI staging pathways versus standard staging pathways, against the consensus reference standard

N Stage	Patient number[a]	WB-MRI staging pathway[b] (n, %)	Standard pathway (n,%)	Difference (95% CI)
N0	107	86 (80)	92 (86)	-6 (-14 to 3)
N1	21	3 (14)	8 (38)	-24 (-49 to 2)
N2	36	20 (56)	26 (73)	-17 (-39 to 5)
N3	20	10 (50)	12 (60)	-10 (-34 to 14)
Overall N stage	184	119 (65)	138 (75)	-10 (-18 to -3)

[a] by consensus reference 3 patients missing data
[b]WB-MRI plus additional generated tests

Appendix Table 8
Per patient agreement for tumour N stage-standard staging pathways versus WB-MRI staging pathways against the consensus reference standard in patients with histological proof of N stage

N Stage	Number of patients[a]	WB-MRI staging pathway[b] (n, %)	Standard staging pathway[c] (n, %)	Difference: WB-MRI[b]-Standard % (CI 95%)
N0	70	56 (80)	60 (86)	-6 (-15 to 4)
N1	19	3 (16)	6 (32)	-16 (-41 to 9)
N2	15	6 (40)	10 (67)	-27 (-61 to 8)
N3	5	3 (60)	3 (60)	0 (-46 to 46)
Overall N stage	109	68 (62)	79 (72)	-10 (-19 to -1)

[a] Patients with histological proof of N stage
[b] WB-MRI plus additional generated tests
[c] 1 patient staged Nx stage by standard staging pathway

Appendix Table 9
Per patient agreement for tumour T stage- WB-MRI staging pathways versus standard staging pathways, against the consensus reference standard

T Stage	Patient number[a]	WB-MRI staging pathway[b,c] (n, %)	Standard pathway[c] (n,%)	Difference agreement: WB-MRI[b] – Standard % (CI 95%)
T1a	29	28 (97)	26 (90)	7 (-9 to 22)
T1b	18	9 (50)	8 (44)	6 (-18 to 29)
T2a	51	20 (39)	23 (45)	-6 (-20 to 8)
T2b	23	8 (35)	9 (39)	-4 (-30 to 21)
T3	33	21 (64)	17 (52)	12 (-9 to 33)
T4	29	13 (45)	17 (59)	-14 (-39 to 11)
Overall T stage	183	99 (54)	100 (55)	-1 (-9 to 8)

[a]4 patients missing data
[b] WB-MRI plus additional generated tests
[c]1 patient each staged Tx stage by WB-MRI and standard staging pathways

Appendix Table 10

Agreement between treatment decisions based on the WB-MRI and standard staging pathways and the retrospective 12-month consensus panel optimal treatment decision

		**WB-MRI staging pathway[b]**		**Standard staging pathway**		**Difference agreement WB-MRI[b] – Standard % (95% CI)**
	Total[a] N	**Agreement n (%)**	**Disagreement n (%)**	**Agreement n (%)**	**Disagreement n (%)**	
All patients	183	152 (83)	31 (17)	151 (82)	32 (17)	1 (-2 to 3)
Patients with metastatic disease	52	29 (56)	23 (44)	28 (54)	24 (46)	2 (-5 to 9)
Patients without metastatic disease	131	123 (94)	8 (6)	123 (94)	8 (6)	0 (-4 to 4)

[a] 4 patients missing at least one type of patient treatment decision
[b] WB-MRI plus additional generated tests

Appendix Table 11

Investigations performed as part of the standard staging pathway

Test	Number of tests (%)[a]
CT neck chest and abdomen	3 (2)
CT chest[b]	8 (4)
CT abdomen and pelvis	11 (6)
CT chest and abdomen	5 (3)
CT chest abdomen and pelvis	2 (1)
PET/CT[c]	181 (97)
CT head[c]	25 (13)
MRI head[e]	16 (9)
TBNA	0 (0)
EBUS/TBNA	6 (3)
EUS	2 (1)
Bone scan	0 (0)
CT guided biopsy	5 (3)
CT liver[f]	6 (3)
Ultrasound guided biopsy	6 (3)
Ultrasound[g]	8 (4)
MRI adrenals	2 (1)
MSK MRI	2 (1)
MRI - Other	1 (1)
X Ray[h]	9 (5)
Other[i]	4 (2)

[a] Patients may undergo more than 1 imaging test

[b] 1 patient had 2 CT chests

[c] 2 patient had 2 PET/CTs

[d] 1 patient had 2 CT heads

[e] 1 patient had 2 MRI heads

[f] 1 patient had 2 CT livers

[g] 1 patient had 2 ultrasounds (transvaginal and abdomen and renal tract)

[h] 1 patient had 3 X-rays

[i] Other tests include 3 bronchial washing and 1 surgical biopsy

Appendix Table 12
Additional Investigations generated by WB-MRI

Test	Number of tests (%)[a]
CT neck chest and abdomen	0 (0)
CT chest	0 (0)
CT abdomen and pelvis	0 (0)
CT chest and abdomen	0 (0)
CT chest abdomen and pelvis	1 (1)
PET CT	26 (14)
CT head	0 (0)
MRI head	6 (3)
TBNA	0 (0)
EBUS/TBNA	4 (2)
EUS	1 (1)
Bone scan	0 (0)
CT guided biopsy	0 (0)
CT liver	0 (0)
Ultrasound guided biopsy	1 (1)
Ultrasound	4 (2)
MRI adrenals	0 (0)
MSK MRI	0 (0)
MRI - Other	2 (1)
X Ray	0 (0)
Other	0 (0)

[a] Patients may undergo more than one imaging test

Appendix Table 13

Number of tests required to complete staging according to the staging pathway

	WB-MRI staging pathway [a] (median test number, 95% CI)	Standard staging pathway days (median test number, 95% CI)	Difference: WB-MRI[a] - Standard (95% CI)
All patients	1<br>(1 to 1)	1<br>(1 to 2)	0<br>(-1 to 0)
Patients with metastatic disease	1<br>(1 to 1)	2<br>(1 to 2)	-1<br>(-1 to 0)
Patients without metastatic disease	1<br>(1 to 1)	1<br>(1 to 1)	0<br>(0 to 0)

[a] WB-MRI plus additional generated tests

Appendix Table 14
Time to complete staging according to staging pathway (95% CI)

	WB-MRI staging pathway (days, 95% CI)	Standard staging pathway (days, 95% CI)	Difference (95% CI)
All patients	13 (12 to 14)	19 (17 to 21)	-6 (-8 to -4)
Patients with metastatic disease	13 (11 to 15)	20 (16 to 23)	-7 (-11 to -3)
Patients without metastatic disease	13 (11 to 14)	19 (16 to 21)	-6 (-9 to -3)

Appendix Table 15
Time to complete staging according to staging pathway-interquartile range

	WB-MRI staging pathway (median days, IQR)	Standard staging pathway (median days, IQR)
All patients	13<br>(7 to 19)	19<br>(13 to 31)

Appendix Table 16
Mean per patent staging cost according to staging pathway

	Frequency (number)			Mean cost per patient (£) (95% confidence limits)	
**Test**	**Conventional imaging**	**WB-MRI**	**Unit cost (£)**	**Conventional imaging**	**WB-MRI**
PET CT	181	26	484	469 (449, 488)	67 (43, 91)
WBMRI	0	187	207	0 (0, 0)	207 (207, 207)
CT head	25	0	120	16 (10, 22)	0 (0, 0)
MRI head	16	6	180	15 (8, 23)	6 (1, 10)
Ultrasound	8	4	52	2 (1, 4)	1 (0, 2)
CT abdomen and pelvis	11	0	112	7 (3, 10)	0 (0, 0)
EBUS/TBNA	6	4	1441	46 (10, 83)	31 (0, 61)
X Ray	9	0	19	1 (0, 2)	0 (0, 0)
CT chest	8	0	97	4 (1, 7)	0 (0, 0)
Ultrasound guided biopsy	6	1	92	3 (1, 5)	0 (0, 1)
CT liver	6	0	97	3 (0, 6)	0 (0, 0)
CT chest and abdomen	5	0	112	3 (0, 6)	0 (0, 0)
CT guided biopsy	5	0	792	21 (3, 39)	0 (0, 0)
CT neck chest and abdomen	3	0	123	2 (0, 4)	0 (0, 0)
CT chest abdomen and pelvis	2	1	123	1 (0, 3)	1 (0, 2)
MRI - Other	1	2	139	1 (0, 2)	1 (0, 2)
EUS	2	1	650	7 (0, 17)	3 (0, 10)
Bronchial washing	3	0	686	11 (0, 23)	0 (0, 0)
MRI adrenals	2	0	180	2 (0, 5)	0 (0, 0)
MSK MRI	2	0	139	1 (0, 4)	0 (0, 0)
Surgical biopsy	1	0	792	4 (0, 12)	0 (0, 0)
Total				620 (574, 666)	317 (273, 361)

Patients may undergo more than one of the same imaging test. Tests ranked by the most frequent across both arms. All costs are 2016/17 UK£. 95% confidence limits derived from 1000 bootstrapped replications of the mean.
